# The quality of life among Otorhinolaryngology residents in Distrito Federal (Brazil)

**DOI:** 10.1590/S1808-86942011000400010

**Published:** 2015-10-19

**Authors:** Gustavo Lara Rezende, Max Sarmet, Ronaldo Campos Granjeiro, Márcio Nakanishi, Carlos Augusto Pires Costa de Oliveira

**Affiliations:** 1 Otorhinolaryngologist. MSc. Student - University of Brasília; 2 Speech and Hearing Therapist. MSc Student - University of Brasília; 3 Otorhinolaryngologist. MSc and PhD Student - University of Brasília; Professor at the Higher School of Health Sciences - DF. Otorhinolaryngologist - Health Secretariat - DF; 4 PhD in Otorhinolaryngology - Medical School - University of São Paulo. Otorhinolaryngologist - University of Brasilia and Health Secretariat - DF; 5 Post-Doctorate - Harvard Medical School, Boston, MA, USA. Head of the Otorhinolaryngology and Head and Neck Surgery - University Hospital - University of Brasília. Universidade Nacional de Brasília

**Keywords:** internship and residency, quality of life, stress, psychological

## Abstract

**Abstract:**

Otorhinolaryngology residents' quality of life must be investigated during medical residency. Work-related factors impacting their lives, such as depression, sleep deprivation and excessive work load may impact the well-being of these individuals.

**Objective:**

To assess and discuss the quality of life of Otorhinolaryngology Resident Physicians in Distrito Federal (Reuni-ORL).

**Materials and Methods:**

Cross-sectional study, the quality of life of each individual was assessed by means of a questionnaire specifically designed for this purpose - Whoqol-bref, proposed by the WHO.

**Results:**

Males had better psychological scores when compared to females (*p*= 0.013). Mean scores comparison among the years in residency were statistically significant only in the psychological domain when comparing the first and second years (*p*=0.046), in which 2nd-year residents had the higher scores.

**Conclusion:**

Despite constant changes to the ENT program (Reuni-ORL) in the Distrito Federal, aiming at improving work and training conditions for residents, there still is a psychological burden in their first year of residency.

## INTRODUCTION

Medical residency is considered the best available method to train medical experts. Nonetheless, aspects associated with it, such as depression, sleep deprivation and an excessive workload, may impact the well-being of these individuals^1^. Defined as a balance between the professional, family, social, physical, mental and financial realms, the quality of life of medical residents require information and further investigations^2^.

Although medical residents are responsible for promoting health in a broader and integral fashion for patients, including not only the physical aspects of it, but also the psychological and the individual's participation in the environment he/she lives, it is known that these professionals not always employ these concepts to their own benefit^3^. There is no evidence about the characteristics which may predict physician's quality of life, not even its impact throughout the medical residency program.

There is no consensus on what quality of life really is. Considering how multidimensional and subjective this concept is, the World Health Organization^4^ created a questionnaire encompassing the physical, psychological, social and environmental aspects associated with this topic. This questionnaire, which is today the basis for studies about individual health status, is a tool used to assess the well-being of resident physicians.

The medical residency program in Otorhinolaryngology of the Federal District has a work load of 60 hours per week, distributed among activities such as outpatient care, surgery, emergency care and classes given in the hospitals certified by the Ministry of Education and Culture (MEC). There are three weekly classes given by the residents, clinical rounds and scientific papers, video-talks and administrative meetings. Residents are required to attend these meetings, as well as the monthly scientific meeting and the programs setup by the ENT Association of the Federal District. The medical resident's pay is regulated by MEC, and set at R$ 1,916.45, at the time this study was carried out. All the residents receive housing grants or free housing; however, there is still no psychological support sponsored by these medical residency programs.

Thus, the assessment of the quality of life of the resident physicians may reveal information regarding the work environment, and point out those situations which need improvements as far as their health is concerned and for excellence in specialist training.

## MATERIALS AND METHODS

This study was carried out encompassing the three Medical Residency Programs (MRP) in Otorhinolaryngology in the Federal District. All the ENT residents from the Federal District took part in the study, i.e. six residents in each program, divided into two resident physicians per year, adding up to three years of specialization program. The inclusion criteria encompassed all the ENT resident physicians in the Federal District. All the participants signed an informed consent form before the study began (Attachment 1).

QL (Quality of Life) assessment for each participant in the study was carried out by means of a questionnaire (Attachment 2) specific for this end, the Whoqol-bref, proposed by the WHO, translated and validated in our country by Fleck (2008)^4^. This questionnaire involves the QL topic by means of the following realms: physical, psychological, environmental and social relations. The questionnaire^4^ is made up of 26 questions. Two questions are general (about QL and health), and the other 24 represent the aforementioned realms. We also collected data about income and family make up, type of housing, how far the resident lived from the hospitals, whether they had their own vehicle, whether they had a pet and the need for psychological or psychiatric support.

The questionnaire was applied in the month of July of 2010, which coincided with the end of the second semester of theoretical-practical activities in the Reuni-ORL-DF program. This is a pioneering teaching program in our country, which brought together the practical and theoretical activities of the three ENT Residency Programs of the Federal District. The questionnaire was applied in the same day for all the participants, in one of the meetings which gathered 18 ENT residents of the Federal District.

The questionnaires were not identified, they were completely anonymous.

The results were evaluated with the help of the SPSS 17.0 Statistics Package. A score was assigned for each answer, and the software yields the total score for each individual, which may vary from 0 to 20. The higher the score, the better the quality of life of the respondent. We analyzed the distribution frequency of the means and standard deviations. We also made a univariate comparison of the means through the t test, calculating the p value, which was < 0.05 for statistical significance.

## RESULTS

All the residents from the three residency programs in ENT agreed to participate in the study and answered the questionnaire. In the Federal District, among the 18 ENT residents, 10 are women and 8 are men. Their mean age was 27(±1.8) years. As far as marital status is concerned, only 4 residents were married.

Most of the residents are from the Federal District (n=5) and Goiás (n=4). The other ones are from Minas Gerais (n=2), Bahia (n=1), Espírito Santo (n=1), Sergipe (n=1), Ceará (n=2), Rio Grande do Norte (n=1) and Mato Grosso (n=1).

Most of the residents rented a place to live (n=11), while the others had their own place (n=4), lived in hos-pital housing (n=2) and only one lived in a student dorm. Their family income is depicted on Table 1.

**Table 1:** Estimated family income of the ENT residents in the Federal District (in R$ × 1,000)

	<5	5-10	10-20
Number of residents	8	6	4

Only three of the residents had lost one of the parents. Most had two siblings (sd=1.1) and six of them had a pet.

When asked if at any time during the residency they had looked for psychiatric or psychological support, only one of the them answered yes.

Most of the residents (sd=0.9) lived in average less than 10 km away from the hospital they worked, and they all had their own car.

### Quality of Life

The “male” residents had a better score insofar as the psychological aspect of the WHO quality of life questionnaire is concerned when compared to their “female” counterparts (*p*=0.01). The marital status was not significantly different in the score. There was no statistically significant difference between the quality of life of the Federal District residents and those from other states.

The first question in the questionnaire (Attachment 2) asked the interviewee how he/she considered his/her quality of life. The only three residents who answered they had a “bad” quality of life were from the third year. Seven residents answered their quality of life was “neither bad nor good”, and eight residents stated they had “good” quality of life.

The second question asked how happy the individual was as far as health is concerned. Three third-year residents answered that they were “not happy” with their health, and two of these were among the three who said they had a “bad” QL. Of the remaining residents, eight answered they were “neither happy nor unhappy”; four said they were “happy about it” and two said they were “very happy”.

**Figure 1:**
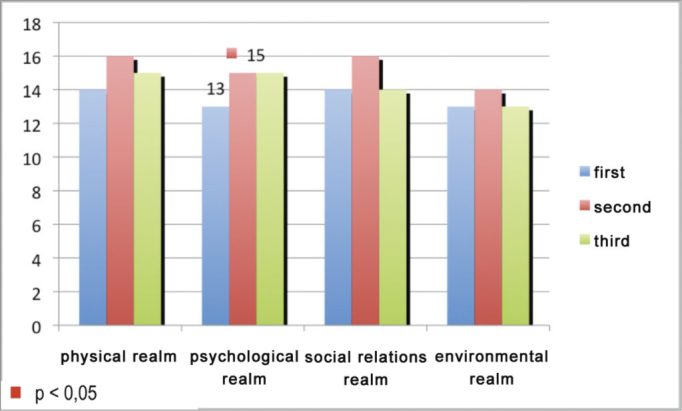
Mean value of the scores corresponding to the realms assessed in the “Whoqolbref” in relation to the year of residency in otorhinolaryngology.

The mean score of the physical, psychological, social and environmental relations of the first (R1), second (R2) and third (R3) year residents are depicted on Figure 1. A comparison of the mean scores between the years of residency was statistically significant only in the psychological realm in relation to the first and second years (*p*=0.04), in which R2 had the highest scores.

Some specific questions in the questionnaire stand out because of the statistically significant difference between the years of residency. In Question 13 (“How available is for you the information you need in your daily routines?”), the R2 scores were higher than the R1 and R3 (*p*=0.02). By the same token, the second-year residents (R2) stated they could concentrate better (question 7) and were happier with their personal relations (question 20) when compared to the R1 residents (*p*=0.01).

In regards of their social relations, there was no statistically significant difference between the residents from each year.

## DISCUSSION

Having more women (55%) in Otorhinolaryngology is different from what was found in the last census (2009) led by the Brazilian Association of Otorhinolaryngology^5^. ENT women represent only 35% of the professionals in this field throughout Brazil. In our study, we did not find statistically significant differences concerning the quality of life between men and women. Franco & Santos^6^ stated that there is a growing number of women surgeons; nonetheless, there still are barriers caused by the lack of institutional support for mothers who are physicians. There were no residents with children in our sample. It may be that the residency is the priority of these women instead of motherhood.

According to regional economic indicators^7^, the cost of life in Brasília is one of the highest in the country. Most of the residents (n=8) had mean family income estimated in less than five thousand Reals. Thus, the basic costs of life of a resident may not have been met by their residency pay only, thus having a negative contribution to the quality of life of these individuals, and preventing them from investing in learning material and professional improvement programs.

The values found in this study's Whoqol-bref are similar to those found among healthy populations^8^, and higher than those found in sick populations^9^, ^10^, ^11^.

We assessed three different moments of the ENT Medical Residency Program. The first year is marked by the adaptation to a new study and work environment^10^.

In this study, most of the residents (n=13) were not from the Federal District, and this was an additional adaptation factor which may negatively impact QL. Moreover, the first year activities in the Reuni-ORL program are more geared towards urgent care - which requires more patience and willingness from the physician to deal with a higher number of patients and situations of urgency/emergency. Esquivel et al.^1^ studied first-year residents in orthopedic surgery and reported that the participants had a greater sensitivity to stress, but did not become emotionally cold, nor distant. On the other hand, Tile et al.^10^ reported that the first-year residents had better rates of mental health, as well as a better general perception of health and social function when compared to second and third year residents. Our study showed that first-year residents had statistically significant lower scores (p=0.04) when compared to second year residents in relation to the psychological realm, which reflects this adaptive period to a new environment and contact with the urgent-care facility.

Third-year residents also suffered an adaptation period to the activities developed in the surgery theater, which require much attention and responsibility; besides having to study more because of the proximity of the Board's Test. Goldin et al.^12^ reported reduction in quality of life and sleep, besides in increase in depression among medical students during their surgery rotations. A study from Raj et al.^13^ detected a significant deterioration in vitality, physical and psychological health when they prospectively studied quality of life associated with health of a group of last-year medical students during ten months. Thus, exhaustion because of the larger number of surgeries in the third year, their entry in the work market and the Board Exams may justify their negative responses insofar as their quality of life is concerned as per asked in the first question of the questionnaire.

The second-year residents, in the middle of this transition, report a better quality of life in numerous aspects. These residents are responsible for fewer patients and surgeries, and they spend less time in the hospital. They have more free time for leisure and to study, and this may explain the higher score in the psychological realm (*p*=0.04) when compared to their first-year counterparts. Alves et al.^3^ mention the stress-reducing strategies during the medical program in their study: to value interpersonal relations and daily life phenomena; a balance between study and leisure; time management; care with one's health, nutrition and sleep; physical activity; religion; to work with one's own personality in order to better cope with adverse situations; search for psychological help.

Results show a worse quality of life among the individuals in the beginning and the at the end of their medical residency program. The Otorhinolaryngology Residency program is one of the most competitive among medical residency programs. Much dedication and hard work are needed in order to take the few spots available in our country. Most of the times, the expectations of the resident when applying to these programs do not correspond to the routine work in ENT. The adaptation to a new environment and the long way before graduating in the specialty often times make these physicians very frustrated, which explains their complaints concerning quality of life in the first year of residency. Among third year residents, there is an expectation of joining the work marked as a specialist, which causes stress, and this may explain the low score in the QL questionnaire.

The present study involved a small sample, and it represents a very specific group of physicians from the Federal District, and it has its methodological limitations. By the same token, the questionnaire was deployed after a quarterly test, which may also have impacted the results. Despite all of this, we used an instrument which has been internationally validated (Whoqol-bref) and has been used in numerous epidemiological surveys involving healthcare professionals, and it is an excellent reference for data comparison.

Despite constant adaptations in the Reuni-ORL ENT residency program of the Federal District, aiming at improving work and study conditions for its residents, there still is psychological stress in the first and third years of residency. New studies, of the cohort type, are necessary in order to better assess the entry of these physicians in the teaching program, so as to cause less negative impact on their quality of life.

## CONCLUSION

The quality of life of ENT resident physicians in the Federal Districts has rates very similar to those of a healthy population. The second-year resident has better quality of life when compared to their counterparts in the first and third years. The first and last year of residency yielded the lowest scores, as far as quality of life is concerned.

**Attachment 1:** Invitation to participate in a study about quality of life

We are doing a study about how people think their lives have been in the past two weeks. We would like to count on your help for a few minutes, to answer a questionnaire. Many questions will be asked about different aspects of your life: your physical health, emotional health, your relationship with friends and family, and your environment.
We would like to make it clear that this study is independent of your work and will not influence in it should you decide not to participate. We assure you that all the information you will provide us will be confidential and will be used only for the purposes of the present study. The disclosure of this information will be anonymous and together with the answers from a group of people.
Should you have any question before you decide, please feel free to ask
Date: ____/____/2010
Interviewee's name: _____________________________________
Signature: _____________________________________________
Interviewer's name: GUSTAVO LARA REZENDE Signature: _________________________________

**Attachment 2:** Abridged questionnaire from the World Health Organization (Whoqol - bref)

**Instructions**						
This questionnaire is about how you feel regarding your quality of life, your health and other aspects of your life. Please, answer all the questions. If you are not sure about which answer to give in any question, please choose among the options, the one which seems more appropriate. Often times, this may be your first choice.						
Please, have in mind your values, aspirations, pleasures and concerns. We are asking what you think of your life, taking the last two weeks as reference. For instance, considering the last two weeks, one question could be:						
		nothing	very little	average	very much	complete
	Do you get from others the support you need?	1	2	3	4	5
You must circle the number which best corresponds to how much support you have got from others concerning your needs in the past two weeks. Therefore, you must circle number 4 if you felt you received “very much” support, as per described below:						
		nothing	very little	average	very much	Complete
	Do you get from others the support you need?	1	2	3	4	5
You must circle number 1 if you feel you received no support at all.						
**Questionnaire**						
Please, read each question, consider what you think and circle the number which seems to be the best answer for you.						
		very bad	bad	not bad, not good	good	very good
1	How do you assess your quality of life?	1	2	3	4	5
		very unhappy	unhappy	not happy, not unhappy	happy	very happy
2	How happy are you with your health?	1	2	3	4	5
The following questions are about how much you have felt some things in the past two weeks.						
		nothing	very little	more or less	very much	Extremely
3	How much do you think your pain (physical) prevents you from doing what you need?	1	2	3	4	5
4	How much do you need some medical treatment for your daily life?	1	2	3	4	5
5	How much do you enjoy life?	1	2	3	4	5
6	How much do you feel your life makes sense?	1	2	3	4	5
7	How much can you concentrate?	1	2	3	4	5
8	How safe do you feel in your daily life?	1	2	3	4	5
9	How healthy is your physical environment (Climate, noise, pollution, attractive things)?	1	2	3	4	5
The following questions are about how completely you have felt or are able to do certain things in these past two weeks.						
		nothing	very little	average	very much	completely
10	Do you have enough energy for your daily life?		2	3	4	5
11	Are you able to accept your physical appearance?		2	3	4	5
12	Do you have enough money to pay for your needs?		2	3	4	5
13	How available are for you the information you need in your day-to-day?		2	3	4	5
14	How much opportunity for leisure do you have?		2	3	4	5
The following questions ask about how well or how pleased you have felt concerning many aspects of your life in the past two weeks.						
		very bad	bad	not bad, not good	good	very good
15	How well are you able to move?	1	2	3	4	5
		very unhappy	unhappy	not happy, not unhappy	happy	very happy
16	How happy are you with your sleep?		2	3	4	5
17	How happy are you with your ability to perform your daily activities?		2	3	4	5
18	How happy are you with your capacity to work?		2	3	4	5
19	How happy are you with yourself?		2	3	4	5
20	How happy are you with your personal relationships (friends, relatives, acquaintances, colleagues)?		2	3	4	5
21	How happy are you with your sex life?		2	3	4	5
22	How happy are you with the support you get from friends?		2	3	4	5
23	How happy are you with the situation of the place where you live?		2	3	4	5
24	How happy are you with your access to healthcare?		2	3	4	5
25	How happy are you with your means of transportation?		2	3	4	5
The following questions refer to how frequent you have felt or experienced certain things in the past two weeks.						
		never	sometimes	often	very often	Always
26	How often do you have negative feelings such as bad mood, despair, stress, depression?	1	2	3	4	5
Did somebody help you fill out this questionnaire?………………………………						
How long did it take you to fill out this questionnaire?……………………………						
Do you have any comments about this questionnaire?…………………………						
THANK YOU FOR YOUR SUPPORT.						
